# Effect of midwife-led care models on maternal and fetal outcomes: A scoping review

**DOI:** 10.6026/973206300210957

**Published:** 2025-05-31

**Authors:** Bama Ramu, Desigamani Kanniyappan, Shankar Shanmugam Rajendran, Revathy Ramasamy, Kannan Kasinathan, Marudhan Anbalagan

**Affiliations:** 1 Department of Obstetrics & Gynecology Nursing, Meenakshi Academy of Higher Education and Research, College of Nursing & Madras Medical College, Chennai, India; 2 Department of Biochemistry, Meenakshi Academy of Higher Education and Research (DU), Enathur, Kanchipuram, Tamil Nadu, India; 3 Department of Pediatric Nursing, College of Nursing, Madras Medical College, Chennai, (The Tamil Nadu Dr. MGR Medical University Chennai), India; 4 Departmrnt of Paediatric Nursing, Meenakshi Academy of Higher Education and Research (DU), College of Nursing & Madras Medical College, Chennai, India

**Keywords:** Midwife-led care, maternal outcomes, fetal outcomes, pregnancy care models, neonatal health

## Abstract

The effect of midwife-led care compared to standard obstetrician-led or shared-care models in improving maternal and fetal outcomes is of interest. Midwife-led care was associated with higher rates of spontaneous vaginal delivery, fewer labor interventions, and greater maternal satisfaction. Neonatal outcomes were comparable or superior, with reduced preterm births and NICU admissions in some studies. Thus, policy support to integrate midwifery models across diverse healthcare systems is essential.

## Background:

The health of mothers and their babies is, and always will be, a major priority for health services globally, which have for decades sought to deliver the best possible pregnancy outcomes while minimizing maternal and neonatal morbidity and mortality [1]. WHO notes that good care -evidence-based woman-centered maternity care is essential for all mothers and newborns to have positive and practical experiences and outcomes? In that context, midwife-led models of care have been around since the national and international debate about whether this is an effective model for improving the health of mothers and babies rates. These models promote continuity of care, continuity of caregivers, and the provision of person-centered, holistic care across pregnancy, labor, birth, and the postnatal period [2]. Despite a global emphasis on more sustainable and affordable ways to deliver maternal health care, the relative effectiveness of midwifery care compared to other models of care delivery is in urgent need of sound evaluation [3]. Despite the lack of standardization in their structure and implementation, midwife-led care models are defined as continuity, relationship-based care, contrasting with obstetrician-led or shared-care models. It emphasizes physiological birth and care that is low in interventions or implements collaborative and shared decision-making. Indeed, it revealed that not only are women much less likely to have a low-intervention labor cesarean, but they also report greater satisfaction following delivery as well when attended by a midwife than when accessed for standard obstetrician care [4]. Thus, whilst more data mustered in favor of midwife-led care has poured in, these findings do not necessarily equate well to the generalizability of high-risk pregnancies, the provision in rural or volume- or resource-challenged settings and as a model suited to pre-existing health systems. To determine whether any midwifery model of care reduces maternal and neonatal adverse outcomes compared with the standard of care for women and babies at four different levels of baseline maternal risk [5]. They discovered that while few systematic conclusions showed benefits to maternal factors (less augmentation of labor or more spontaneous vaginal birth), more discussed long-lasting neonatal conditions are seen more conspicuously in complicated pregnancies. However, the skepticism seen elsewhere in the midwifery models of care literature has not stayed behind, at least not for the situations that do not permit the scaling up of midwife-led models, particularly regarding equivalence for access to maternal health care.

The push for midwife-led care in many parts of the world reflects a global search for good quality, cost-effective maternity care. The potential benefits of quality midwifery, particularly in settings where access to a sufficient healthcare workforce is not a reality, have led countries to consider the significant scale-up of midwifery to address care needs during childbirth for mothers and newborns [6]. Recognizing that midwifery can play a good role in improving maternal and perinatal outcomes and reducing the burden of obstetricians and the overall maternity service, the WHO has advocated reinforcing midwifery services. However, midwife-led care may only be effective if it occurs within the context of a particular health service system, a well-trained workforce practicing within an appropriate regulatory system and culture in which midwifery practice is accepted [7]. As the types of midwifery differ from one healthcare system to another, we will conduct a scoping review to identify literature worldwide addressing the impact of midwifery care on maternal-fetal outcomes. This review aims to summarise the amount of research on midwifery care models, the maternal and fetal health outcomes influenced by these models, and the absence of research to fill the gaps that prevent a comprehensive answer to the question of the best model of midwifery care to increase the chances of positive maternal and fetal health outcomes beyond completion of pregnancy. Maternal (mode of delivery, labor interventions, maternal satisfaction, and post-partum complications) and fetal outcomes (birthweight, Apgar scores, NICU admissions, and perinatal mortality) would be specific target outcomes of the scoping review. Therefore, it is of interest to describe the global research landscape regarding midwifery models and their associated maternal and fetal outcomes to inform best practices and highlight areas in need of further investigation.

## Methodology:

This scoping review followed the Arksey and O'Malley framework and adhered to PRISMA-ScR guidelines. The objective was to explore the impact of midwife-led care models on maternal and fetal outcomes. A systematic search was conducted across databases including PubMed, Cochrane Library, Scopus, CINAHL, Web of Science, and Google Scholar using key terms like "midwife-led care," "maternal outcomes," and "fetal outcomes." Studies included randomized controlled trials, cohort, case-control, and qualitative studies involving pregnant women receiving midwife-led care. Titles and abstracts were screened by two reviewers, with full-text analysis for eligible articles. Data were extracted using a standardized form covering study characteristics, population details, type of midwifery care, and outcomes. Maternal (*e.g.*, mode of delivery, complications) and fetal (*e.g.*, birthweight, NICU admission) outcomes were assessed. The results were narratively synthesized without quality assessment, as is typical for scoping reviews, and findings were thematically grouped for analysis.

## Results:

A narrative synthesis was used to describe the findings, highlighting limitations in existing literature and recommendations for future research.

## Quality assessment:

Individual studies were not quality assessed as this was a scoping review. Instead, the limitations of each study were explored in terms of their overall contribution to the evidence base.

## Review:

The effectiveness of midwife-led care models has been the subject of increasing research, particularly in low-risk pregnancies. Evidence suggests that midwife-led care may significantly improve maternal and fetal outcomes compared to traditional obstetrician-led care. This review synthesizes findings from several recent studies, shedding light on the impact of midwifery-led models on various aspects of pregnancy, childbirth, and maternal well-being.

## Midwife-led care versus obstetrician-led care:

Sethi *et al.* (2024) conducted a massive systematic review and meta-analysis of over 1.4 million pregnancies between midwife-led cares versus obstetrician-led care for low-risk pregnancies. The study found that midwife-led models were linked to a decrease in cesarean deliveries as well as a decrease in interventions such as the use of epidurals or forceps. Women in midwife-led care were also more likely to be satisfied with their childbirth experience. These findings point to the unique benefits of midwife-led care, which tends to be a more natural, less medicated approach to childbirth that can lead to better outcomes for mothers and babies [8].

## Impact on birth outcomes:

Examining the way-midwifery-led care might improve birth outcomes among low-risk-pregnant women (Ernawati, 2024). Skilled-attendant-led care was linked to higher odds of spontaneous vaginal births and lower rates of preterm birth. Similarly, Fitriana *et al.* (2024) [21] conducted a meta-analysis assessing the effect of midwife-led care on the mode of birth. Their results echoed Ernawati's findings that midwife-led care significantly reduced cesarean rates and enabled safer, less clinically influenced delivery processes [9]. Moreover, Hua *et al.* (2022) highlighted that midwife-led continuity of care, which involves continuous care throughout the pregnancy, labor, and postpartum periods, significantly increased women's satisfaction with antenatal, intrapartum and postpartum care. The study in Ethiopia demonstrated that such care models could lead to more positive birth experiences and improved maternal mental health outcomes [10].

## Quality of care and maternal mental health:

Research by Hua *et al.* (2023) provides an in-depth analysis of the psychosocial benefits of midwife-led care, highlighting that it is effective in managing birth outcomes, as well as influencing maternal mental health [10]. Kuipers *et al.* reported fewer cases of postpartum depression and anxiety among women receiving midwifery-led services [11]. They presented mixed-methods synthesis that emphasized the positive effects of midwife-led care on maternal mental health .They theorized that such models would benefit maternal mental health and well-being, providing insight into the need for continuity in care.

## Challenges and areas for improvement:

While the evidence strongly supports the benefits of midwife-led care, challenges remain in its broader implementation. Page *et al.* (2024) conducted a concept analysis to define midwifery-led care in the United States, highlighting the variability in how such care is conceptualized and applied. Their study stressed the importance of clear definitions and frameworks to ensure consistency and effectiveness in midwifery practice, especially as it becomes more integrated into the broader healthcare system [12]. Additionally, logistical and systemic barriers to implementing midwife-led care models-such as healthcare policies, training, and access to resources-remain obstacles that need addressing to maximize the potential benefits of midwifery-led care across diverse healthcare settings.

## Discussion:

The body of research on midwife-led care continues to grow, with an increasing number of studies examining its impact on maternal and fetal outcomes. The studies reviewed here highlight several key aspects of midwifery-led care, including the satisfaction of women receiving care, the effect on birth outcomes, and the comparison of midwifery care with traditional obstetrics models. The findings suggest that midwife-led models, particularly those emphasizing continuity of care, offer significant benefits to both mothers and babies, although challenges and areas for improvement remain.

## Maternal satisfaction and continuity of care:

Perhaps the most consistent finding from the studies is that women receiving midwifery-led care report higher satisfaction. Hua *et al.* (2022) across antenatal, intrapartum and postpartum stages are comparing antenatal, intrapartum and postpartum satisfaction. This is consistent with previous literature that has suggested that a relationship where the midwife knows the woman well over time creates a supportive environment to promote a positive experience of pregnancy [10]. Furthermore, midwife-led care enables continuity, promoting better communication, individualized care, and a lower-stress environment for mothers-to-be. This matters because research has found that mothers who feel positively about their birth will likely have better physical and mental health outcomes in the months and years to come. Some publications hypothesize this, such as the research of Hua *et al.* (2022), which reinforces that midwifery is not only a clinical model, as midwife care can positively affect the psychosocial well-being of women during pregnancy.

## Birth outcomes:

## A comparison with obstetrics models:

The study also confirmed the effect of midwifery-led care on birth outcomes. Alcaraz-Vidal *et al.* (2024) and Beckingham *et al.* (2022) contribute to a growing body of evidence associating midwife-led care with favorable maternal and neonatal outcomes. For instance, Alcaraz-Vidal *et al.* a midwifery-led and first in a high-complexity public hospital in Spain, showed that these models are not unsafe-ed for maternal or neonatal health [14, 15]. Women cared for in this unit were similar to or better than those receiving care in traditional obstetrics services concerning complications and intervention rates. This is in keeping with earlier meta-analyses' findings that midwifery-led care is associated with fewer medical interventions (cesarean sections) and fewer preterm births. Similarly, Beckingham *et al.* (2022) showed that the implementation of professional midwife-led maternity care in India improved maternal and fetal outcomes in healthy pregnant women, confirming the potential of this model to reduce maternal and neonatal morbidity and mortality.

## Postpartum care and emergency department utilization:

Sorbara *et al.* (2024) compared the postpartum emergency department (ED) use after the midwifery model to obstetrics-model care. They found that women who received midwifery-led care were less likely to visit the emergency department. This may imply that the continued involvement of a midwife in a woman's postnatal care and the more individualized approach offered by a midwife might lead to less requirement for the need for emergency postnatal interventions, which is a significant factor in developing maternal morbidity and mortality throughout the postpartum period. The lower rates of postpartum complications and emergency visits are consistent with earlier research showing that midwifery-led models may be able to avert complications that warrant emergency treatment, in part because midwives tend to emphasize preventive care and monitoring, which can prevent complications from developing to the point that they require emergency procedures. Sorbara *et al.*'s results indicate that the bacteria can also be at full length at all these sites. (2024) are especially relevant to the management of healthcare resources. There is often a lot of pressure on emergency care systems, notably in the limited healthcare infrastructure setting. The decrease in ED visits by women who received midwifery care indicates that these models not only provide better health outcomes to women. Still, it may also relieve pressure on emergency care systems. Such a benefit would be relevant in high- and low-resource settings [16].

## Challenges and considerations:

Even though evidence for the effectiveness of midwife-led care is strong, the challenges of implementing this model must be addressed. In some settings, integrating midwifery-led care into existing health systems remains challenging. Page *et al.* (2024) reflect variability in the definition and implementation of midwifery-led care throughout the United States and emphasize the need for more explicit midwifery-led frameworks and a better understanding of midwives' role in maternal care. Existing regulatory frameworks with clear definitions and firm policies are necessary to facilitate the process of integrating midwifery-led models of care into the healthcare system, especially in portions of the healthcare system where obstetricians are the primary care providers [12, 13]. In addition, although midwife-led care is effective, it may not be appropriate for all women, especially those with high-risk pregnancies. The most comprehensive picture of care may be through a collaborative model that integrates midwifery and obstetric care together. This may also guarantee that women with higher risk profiles receive specialized care while having the benefit of continuity of care and personal care from the midwives. Fikre *et al.* conducted a systematic review and meta-analysis (2023) that demonstrated an association between MLC and improved pregnancy outcomes, evidenced by lower rates of PTB, stillbirth, and low birth weight. All related evidence supports midwife-led interventions that can impact maternal and neonatal health, and these are especially important in resource-limited settings where access to obstetrician-led care may be constrained [17]. Ota *et al.* the impact of antenatal interventions, including those for midwives, has been shown to prevent stillbirth and perinatal mortality. Their detailed analysis of Cochrane systematic reviews shows that evidence-based and structured midwifery care interventions are essential in decreasing the risks of poor pregnancy outcomes [18]. Bagheri *et al.* performed a meta-analysis (2021) to build on this knowledge by showing how maternal satisfaction is substantially greater with MLC and medical interventions (*e.g.*, cesarean section), and neonatal outcomes are improved compared to the standard model of care. Such decision-making results demonstrate that MLC models are holistic in delivering patient-centered care, which is thought to improve perinatal health indicators [19]. More recent research by Sriram *et al.* (2024) emphasizes the scalability and effectiveness of midwife-led care, especially for low-risk pregnancies. Their systematic review and meta-analysis of > 1.4 million pregnancies showed that it was associated with lower rates of medical induction and instrumental deliveries without compromising neonatal safety. These findings question the prevailing practice of an obstetrician-model care pattern for all pregnancies and propose that MLC may be an effective and safe alternative, especially in low-risk cases [20]. Fitriana *et al.* have similarly systematically assessed the effect of MLC on the mode of birth (2024), which showed midwife-led care to be associated with increased spontaneous vaginal birth while reducing the risk of cesarean delivery. Their review describes the potential of MLC to enhance physiological childbirth and reduce unnecessary medical interventions while supporting maternal autonomy in decision-making [21].

## Conclusion:

Midwifery-led care models significantly improve maternal and neonatal outcomes while minimizing medical interventions. They offer psychosocial and economic advantages that support maternal well-being within global health systems beyond clinical benefits. Therefore, it is important to further explore integrated models combining midwife-led and obstetrician-led care to ensure safe, personalized maternity services, particularly for high-risk pregnancies.
